# ACGAN for Addressing the Security Challenges in IoT-Based Healthcare System

**DOI:** 10.3390/s24206601

**Published:** 2024-10-13

**Authors:** Babu Kaji Baniya

**Affiliations:** Department of Computer Science and Information Systems, Bradley University, Peoria, IL 61625, USA; bbaniya@fsmail.bradley.edu

**Keywords:** accuracy, ensemble, predictive, discriminator, generator, IoMT, healthcare

## Abstract

The continuous evolution of the IoT paradigm has been extensively applied across various application domains, including air traffic control, education, healthcare, agriculture, transportation, smart home appliances, and others. Our primary focus revolves around exploring the applications of IoT, particularly within healthcare, where it assumes a pivotal role in facilitating secure and real-time remote patient-monitoring systems. This innovation aims to enhance the quality of service and ultimately improve people’s lives. A key component in this ecosystem is the Healthcare Monitoring System (HMS), a technology-based framework designed to continuously monitor and manage patient and healthcare provider data in real time. This system integrates various components, such as software, medical devices, and processes, aimed at improvi1g patient care and supporting healthcare providers in making well-informed decisions. This fosters proactive healthcare management and enables timely interventions when needed. However, data transmission in these systems poses significant security threats during the transfer process, as malicious actors may attempt to breach security protocols.This jeopardizes the integrity of the Internet of Medical Things (IoMT) and ultimately endangers patient safety. Two feature sets—biometric and network flow metric—have been incorporated to enhance detection in healthcare systems. Another major challenge lies in the scarcity of publicly available balanced datasets for analyzing diverse IoMT attack patterns. To address this, the Auxiliary Classifier Generative Adversarial Network (ACGAN) was employed to generate synthetic samples that resemble minority class samples. ACGAN operates with two objectives: the discriminator differentiates between real and synthetic samples while also predicting the correct class labels. This dual functionality ensures that the discriminator learns detailed features for both tasks. Meanwhile, the generator produces high-quality samples that are classified as real by the discriminator and correctly labeled by the auxiliary classifier. The performance of this approach, evaluated using the IoMT dataset, consistently outperforms the existing baseline model across key metrics, including accuracy, precision, recall, F1-score, area under curve (AUC), and confusion matrix results.

## 1. Introduction

HMSs are integrated platforms designed to streamline clinical examination details, medical history, medication timelines, treatment plans, immunization or vaccination records, health professional and provider information, and overall costs related to healthcare services [1]. The information technology (IT) infrastructures extend these services to remote locations, marking a significant milestone in HMS development. This technological integration enables improvements in patient care, enhances the efficiency of healthcare services, and facilitates the storage and retrieval of patient and healthcare-provider information [2]. The use of IT in HMSs contributes to timely decision-making by healthcare professionals [3]. Notably, HMSs support the early detection of health issues in patients, empowering healthcare professionals to intervene proactively [4]. It fosters improved care coordination, promoting a shift towards preventative and predictive healthcare, and facilitates efficient resource allocation. With the ability to tailor treatment plans based on detailed patient data, the HMS plays a pivotal role in personalized healthcare [5,6].

HMSs help to connect individuals residing in geographically disadvantaged rural areas and urban areas facing challenges like existing health conditions, aging, or mobility issues that hinder access to basic healthcare services [7]. IoT acts as a mediator, establishing seamless connections between patients and healthcare service providers. With the increasing aging population, IoT-based healthcare emerges as an essential tool to deliver convenient medical services to vulnerable individuals in communities. The COVID-19 virus spread worldwide rapidly, and it instilled fear and anxiety not only among the general population but also among healthcare professionals and service providers, with people canceling or postponing regular checkups in clinics and hospitals due to concerns about the virus’s spread. The heightened fear and anxiety not only deterred individuals with existing healthcare conditions from seeking immediate medical attention but also led to the cancellation of appointments [7]. In response to this challenging situation, a remote healthcare monitoring system emerges as an effective solution, facilitating the delivery of services to patients and healthcare providers with less fear of virus transmission. Despite the numerous challenges, implementing such a system creates a win–win situation for both patients and healthcare service providers. Undoubtedly, the COVID-19 pandemic was a contributing factor to the exponential surge in IoT-based healthcare systems.

Recently, IoT has emerged as an important domain, particularly contributing to the advancement of HMSs. The primary goal of IoT-based HMSs is to precisely monitor individuals and establish connections between various (healthcare-related) services and entities globally via the Internet [8]. This facilitates the collection, sharing, monitoring, storage, and analysis of the data generated by these entities [9,10,11]. The advent of technologies such as the IoT, machine learning (ML), and deep learning (DL) has ushered in a new paradigm. This paradigm involves the interconnection of physical objects in intelligent applications like smart cities, smart homes, smart grids, smart vehicular systems, and smart healthcare, enabling remote addressing and control [12]. An IoT-based remote monitoring healthcare system holds significant importance in diagnosing disorders and monitoring patients for effective medical care. The integration of sensor networks into the human body proves immensely valuable in facilitating these healthcare endeavors [13].

The integration of IoT in healthcare signifies a breakthrough, presenting both opportunities for remote health services and notable challenges. The promising aspects include accessing reliable, convincing, and cost-effective services from remote healthcare professionals and providers. IoT systems have enabled the construction of reliable HMSs using affordable and low-power sensors [1]. However, the challenges faced by IoT mirror those encountered in HMSs. A significant hurdle involves managing the vast array of data formats generated by IoT devices, which encompass wearable sensors (blood oxygen saturation (SpO2) sensors, blood pressure sensors, temperature sensors, and electrocardiogram (ECG) sensors, etc.), medical implants, and monitoring equipment [1,14,15,16]. These devices continuously collect data about patients’ health status. Another critical challenge lies in ensuring the security and privacy of these data. Given the sensitive nature of healthcare-related information, safeguarding patient details, diagnosis reports, medication plans, and the privacy of healthcare professionals remains a paramount concern [16,17].

**Figure 1:**
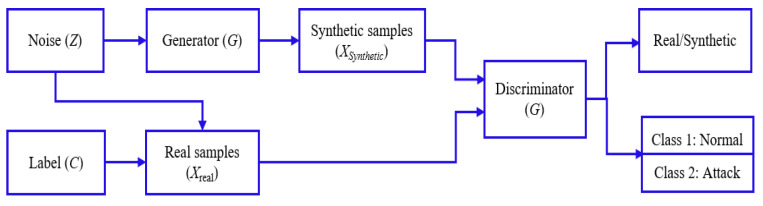
ACGAN architecture: label (*C*), noise (*Z*), real samples ($X_{real}$), generator (*G*) synthetic samples ($X_{synthetic}$), discriminator (*D*), and predicated classes: ‘Normal’ and ‘Attack’.

To tackle this challenge, we employed the ACGAN, a powerful DL model known for generating novel data that closely resemble the training dataset. Unlike conventional GAN paradigms, ACGAN incorporates an auxiliary classifier within the discriminator. This classifier enhances the prediction of class labels for both real and synthetic samples, adding an additional layer of information [18,19]. This approach enables the discriminator to distinguish between real and synthetic samples and classify them into predefined categories (Normal and Attack), as shown in Figure 1. Similarly, the generator aims to produce samples that the discriminator will recognize as real and the auxiliary classifier will correctly classify into the known class. This additional role helps the generator focus on producing high-quality, class-specific samples (either Normal or Attack samples in IoMT dataset). Given the highly imbalanced distribution of the WUSTL-EHMS-2020 dataset (https://www.cse.wustl.edu/~jain/ehms/index.html), (accessed on 22 November 2023), with 87.4% of samples categorized as Normal and the rest as Attack, there is a risk of bias toward the majority class. ACGAN addresses this by creating highly representative samples of the Attack categories, leveraging its objective function to optimize performance. The stability of training is another area in which ACGANs offer improvements. The additional classification task acts as a form of regularization, providing more structured feedback to both the generator and the discriminator. This structure can lead to more stable training and faster convergence, addressing some of the instability issues commonly associated with GAN training. The enriched feedback from the auxiliary classifier helps both networks learn more robustly and efficiently [20,21].

The WUSTL-EHMS-2020 dataset has two different features sets, biometric and network flow metrics, as shown in Table 1. First, a thorough investigation was conducted into the role of both feature sets in intrusion detection in the IoMT. The findings indicate that despite having only eight biometric features (one-third the number of network flow metric features), the biometric set exhibits significant discriminative ability, performing nearly as effectively as the network flow metrics. The performance results are summarized in experimental result section. Second, this method addresses the issue of imbalanced datasets and mitigates classifier bias toward the majority class by generating synthetic samples for the minority class using GAN. This approach effectively reduces bias and enhances model robustness. Furthermore, *k*-fold cross-validation, dropout, and early stopping were incorporated to mitigate the overfitting issue, particularly due to the large number of synthetic samples in the ‘Attack’ category. Third, a comparison was made between the results of the generation of synthetic samples using GAN and the Synthetic Minority Over-sampling Technique (SMOTE). The comparison revealed that GAN produces more realistic and effective samples than SMOTE, further enhancing the model’s performance.

### Contributions

- Exploration of IoMT Attack Detection: The study explores the ability of biometric and network flow metric feature sets to detect attacks in the IoMT. Despite the biometric feature set being quantitatively smaller (approximately one-third of the network flow metric set), it demonstrates a higher discriminability, with an attack detection rate of 0.966.
- Three Evaluation Methods: The evaluation was conducted in three ways: using biometric features, network flow metrics, and a combination of both feature sets.
- Generation of Synthetic Samples: To minimize bias toward the majority class, two distinct approaches, ACGAN and SMOTE, were employed to generate synthetic samples. Their comparative evaluation is presented in this article.
- Comparison with Baseline Method: The results were compared with the baseline method [1], showing that the proposed method achieves a higher attack detection rate.

This paper is structured as follows: Section 2 presents the multifaceted challenges encountered in HMSs. Section 3 offers an overview of EHMSs. Subsequently, Section 4 provides details on the dataset used, and Section 5 encompasses the experimental details, discussions of the proposed method, and the key findings. Section 6 presents a comparative analysis with existing methodologies. The conclusion in Section 7 presents a succinct and insightful summary of the proposed method, outlines potential future directions, and highlights areas for future work.

## 2. Challenges in Healthcare Monitoring Systems

**Figure 2:**
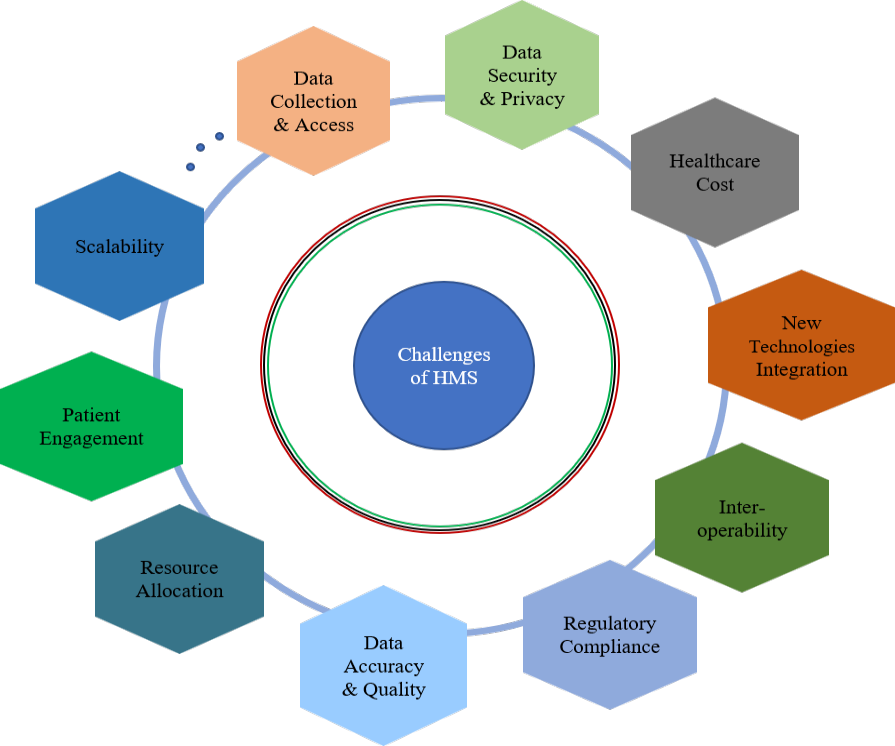
The challenges of healthcare monitoring systems.

The rapid growth of HMSs in healthcare faces multifaceted challenges that impact their functionality and overall effectiveness in delivering the services, as shown in Figure 2. Their cost poses perhaps the most significant challenge for current healthcare service providers aiming to successfully implement a remote patient monitoring system. This challenge introduces additional costs for healthcare service providers, which can be broadly categorized into three areas: equipment purchases, servicing, and monitoring expenses [22]. The costs are inherently additive, especially during the installation of new technologies in facilities. This process not only incurs expenses regarding the technology itself but also demands additional investments in staff and technician training on the remote healthcare monitoring system. Hummel et al. designed an economic model to compare outcomes with and without a remote monitoring system. Their findings revealed a decrease in hospitalization costs and an increase in life expectancy with the implementation of a remote health monitoring system. This evidence suggests that remote monitoring systems prove to be cost-effective when compared to the absence of such systems [23].

### Challenges Components

The global healthcare system fundamentally aims to enhance the delivery of high-quality healthcare services. Identifying the determinants of quality poses a complex challenge due to a myriad of variables. ‘‘Quality” itself is a somewhat elusive term that proves difficult to precisely define [24]. According to the European Commission and the Institute of Health, quality defines healthcare that is effective, safe, and responsive to the needs and preferences of patients [25]. Recognizing the determinants of compliance holds the potential to enhance regulatory processes and provide valuable insights for quality improvement initiatives undertaken by healthcare service providers and policymakers [24]. Regulation serves as a response to the variability in quality within various sectors. The authorities establish a set of norms or standards to serve as benchmarks for quality, and subsequently evaluate the extent to which healthcare organizations and individuals adhere to these established standards [26].

The integration and interoperability of new technology: Integrating new technology is always a challenging task with a direct impact on patient care, throughput, patient safety, seamless connection, reduced human intervention, and the overall perception of remote healthcare systems. Barriers to the integration of such technology include the need to design new layouts and operations for the technology, along with the need to decluster and determine equipment positions, among other factors [27]. The lack of protocols, platforms, and standardized technologies across different healthcare systems leads to serious interoperability issues [7,28]. This hinders the seamless exchange of different data formats and places constraints on processing capabilities, which are particularly critical in healthcare, where real-time monitoring and decision-making are of the utmost importance and can have severe consequences, including adverse outcomes [29,30]. Interoperability is defined as the ability to acquire data or records from various vendors and to interact seamlessly with other healthcare-provider computers across local or wide-area networks, irrespective of their physical architecture and operating systems. This is feasible through hardware and software components that conform to open standards, much like those employed for the internet [31].

Security: The role of secure healthcare data is important in making informed decisions and fostering patient trust in the context of the IoMT. Therefore, security vulnerabilities have emerged as a noteworthy concern in both the software industry and the realm of cybersecurity, indicating a need for further enhancements in current vulnerability detection approaches [11,32,33]. Safeguarding patient data from unauthorized access, modification, or breaches stands as another formidable challenge, especially with the surging volume of Electronic Health Records (EHRs) intensifying cybersecurity threats [14,34]. IoT systems are typically structured into four layers: the application layer, the middleware or support layer, the networking and data transmission layer, and the perception or sensing layer. Each of these layers employs different technologies and presents unique security challenges. Common IoT-related risks include denial-of-service attacks, spoofing, jamming, eavesdropping, data manipulation, and man-in-the-middle attacks [35].

The accuracy and quality of healthcare data are paramount for informed decision-making. Issues such as duplicates, errors, and inconsistencies compromise the integrity of information within the healthcare domain [36,37]. In system, ensuring data quality involves a comprehensive consideration of various factors. This encompasses evaluating the entire lifecycle of health data, addressing issues stemming from errors and inaccuracies within the data, understanding the source(s) and history of the data, and acknowledging how the underlying purpose of data collection influences the subsequent analytic processing and the knowledge expected to be derived from the data [37].

In the healthcare system, duplicate records may arise during technical analysis and administrative processes, such as errors in entering patient information or in the integration of patient data from different information systems [38]. According to Erel et al., the estimated cost associated with a single pair of duplicate records contributes to the financial burden for both patients and service providers [36].

Data collection in healthcare involves systematically capturing pertinent information from various stakeholders, including patient details, tests and diagnostic results, medication plans, medical history, and the current status of patients. The objective is to gather these data in real-time, enabling healthcare professionals to access information promptly. This real-time access proves invaluable in emergency situations, allowing healthcare professionals to swiftly retrieve data and take immediate actions based on the patient’s current situation [15].

Efficient resource allocation, diverse equipment, and proper facilities are crucial for effective healthcare services. Inaccurate projection and the inaccurate allocation of resources can lead to inefficiencies, adversely impacting patient care and imposing financial burdens on patients [14]. Active patient engagement in remote healthcare systems and treatment plans poses a formidable challenge, especially among patients, particularly the older generation, who are accustomed to traditional healthcare systems. Encouraging patients to actively participate in their healthcare journey and raising awareness requires overcoming established norms and fostering a new culture of engagement [39].

## 3. Enhanced Healthcare Monitoring Systems

**Figure 3:**
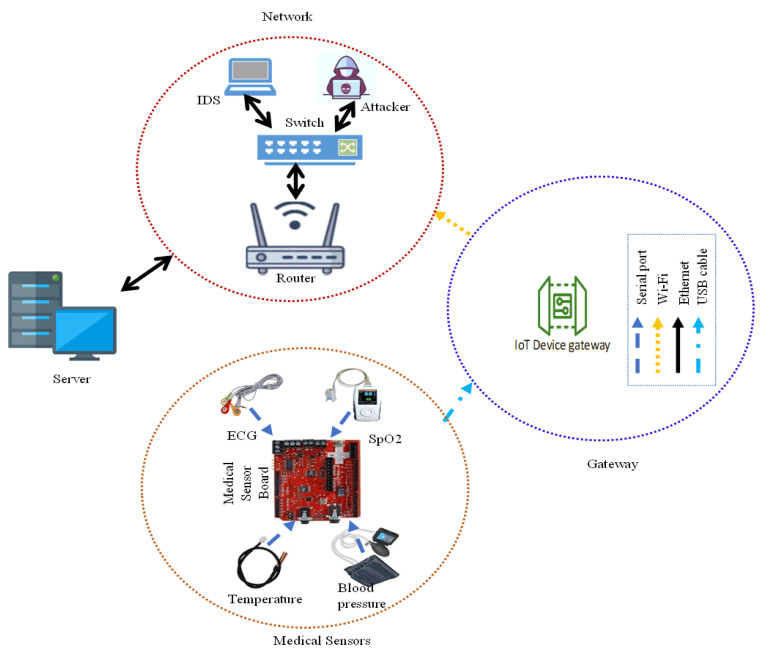
Overview of EHMS: medical sensors, gateway, network (router, switch, attacker, intrusion detection system), and server [1].

The EHMSs, depicted in Figure 3, comprise a medical sensor board that is responsible for collecting patient data from various sensors, including a temperature sensor, blood pressure sensor, SpO2 sensor, and ECG sensor, which are all strategically placed on the patient’s body [1]. The data traverse the network, passing through a gateway and switch, on their way to the server. During the data transmission process, there exists a potential vulnerability where attackers may exploit the weakest point to intrude, spoof, or alter the original data before reaching the server, a scenario commonly referred to as a man-in-the-middle attack [23,24]. To mitigate such threats, an intrusion detection system (IDS) is integrated into the EHMS. This IDS actively captures both network and patient data. The captured data undergo processing within the IDS for both training and testing purposes to detect any potential malicious activities [40,41].

## 4. Dataset Description

The EHMS dataset was recently collected in 2020 at Washington University in St. Louis (WUSTL). This structured dataset provides a rich resource for healthcare monitoring and analysis, offering insights into network-related features and individual-specific biometric characteristics. The dataset encompasses a total of 16,318 samples, categorized into two primary types: ‘Attack’ and ‘Normal’. Statistically, the dataset is characterized by an imbalance, with the majority of samples labeled as Normal, constituting approximately 87.46%, while 12.54% are identified as Attack samples. The dataset comprises 43 distinct features, which are further classified into two main categories: network flow metric (totaling 35 features) and biometric features (8 features). In the network flow metric, some irrelevant features, such as the MAC address and gateway-related attributes, were removed (and were not considered in the performance evaluation). Network flow features play a crucial role in understanding network behavior and patterns, while the dataset includes a smaller set of biometric features offering insights into individual-specific characteristics, contributing to a more comprehensive understanding of healthcare-related activities. Table 1 presents the feature names, their descriptions, and their corresponding categories. Similarly, the correlation coefficient was calculated for both feature sets (network flow and biometric) separately. The experiment revealed that certain network flow features, such as DstLoad, DstGap, SrcGap, Trans, DIntPktAct, and dMinPktSz, have an insignificant correlation coefficient. Consequently, these features were discarded and not included in the validation of the proposed model.

## 5. Experimental Results

The experimental setup was designed to evaluate the IoMT attack detection ability in two phases (the same as the feature distribution). In the first phase, the performances of two sets of features (biometric and network) were measured individually to determine which set is more effective for IoMT attack detection and protecting against the possible loss or theft of medical and patient data. In the second phase, both feature sets were combined, and the performance metrics were measured using ACGAN. The goal was to assess their combined effectiveness and strength in detecting IoMT attacks compared to the individual feature sets. The outcomes of the classifier (ACGAN) were meticulously calculated using specific formulas. The evaluations (accuracy, precision, recall, and F1-score) were based on four distinct parameters: true positive (TP), representing instances when the system correctly detected attacks in the dataset; true negative (TN), denoting cases where the system correctly identified the absence of attacks; false positive (FP), indicating instances where the system wrongly detected attacks in their absence in the IoMT dataset; and false negative (FN), representing cases where the system failed to detect attacks when the risk was present in the dataset. This comprehensive evaluation provided a detailed understanding of the overall performance of each classifier in handling Normal and Attack cases based on these matrics.

1. Accuracy estimates the ratio of recognized risk for all conditions (cases). If accuracy is higher, the machine learning model is better.
  (1) $$Accuracy=\frac{TP+TN}{TP+TN+FP+FN},$$
2. Precision measures the accuracy of the model in predicting positive instances. High precision indicates that when the model predicts a positive class, it is likely to be correct.
  (2) $$Precision=\frac{TP}{TP+FP},$$
3. Recall is the ratio of true positive predictions to the total number of actual positive instances. This calculates the ability of the model to capture all positive instances.
  (3) $$Recall=\frac{TP}{TP+FN},$$
4. The F1-score is a metric that combines both precision and recall. It is the harmonic mean of precision and recall and provides a balanced measure of a model’s performance.
  (4) $$F1-\mathrm{score}=2\times \frac{\mathrm{Precision}\times \mathrm{Recall}}{\mathrm{Precision}+\mathrm{Recall}}$$

### ACGAN

In the ACGAN, every generated sample has a corresponding class label, *c*, in addition to the noise (*z*). The discriminator (*D*) outputs two probability distributions: one over the sources, P(S|X), which indicates whether the sample is real or synthetic, and one over the class labels, P(C|X), which classifies the sample into one of the predefined classes [42]. The objective function of ACGAN is as follows:

1. Log-likelihood of the correct source ($L_{S}$): The objective function ensures that the discriminator correctly identifies whether the sample is real or synthetic.
2. Log-likelihood of the correct class ($L_{C}$): This ensures that the discriminator correctly classifies the sample into its respective class.

During the training of ACGAN, the loss function of discriminator (*D*) is expressed as follows:

(5) $$L_{D}=L_{S}+L_{C}$$

where

(6) $$L_{S}=E\left[\log P\left(S=\mathrm{real}|X_{\mathrm{real}}\right)\right]+E\left[\log\left(1-P\left(S=\mathrm{synthetic}|X_{\mathrm{synthetic}}\right)\right)\right]$$

and

(7) $$L_{C}=E\left[logP\left(C=c|X_{real}\right)\right]+E\left[logP\left(C=c|X_{synthetic}\right)\right]$$

The loss function of the generator (*G*) is given as follows:

(8) $$L_{D}^{\prime }=L_{C}^{\prime }-L_{S}^{\prime }$$

where

(9) $$L_{C}^{\prime }=E\left[logP\left(C=c|X_{synthetic}\right)\right]$$

and

(10) $$L_{S}^{\prime }=E\left[logP\left(S=synthetic|X_{synthetic}\right)\right]$$

The proposed method utilizes the ACGAN to enhance performance, as outlined in Algorithm 1.
**Algorithm 1** Auxiliary Classifier Generative Adversarial Network (ACGAN) 1:Initialize the generator *G*, discriminator *D*, and auxiliary classifier *C* with random parameters 2:Set the number of training iterations *N* 3:**for** each training iteration i=1 to *N* **do** 4:  // **Step 1: train discriminator and auxiliary classifier** 5:  Real data samples *x* and corresponding labels *y* 6:  Noise *z* and corresponding labels $y_{\mathrm{synthetic}}$ 7:  Generate synthetic data samples $\tilde{x}=G\left(z,y_{\mathrm{synthetic}}\right)$ 8:  Update *D* by maximizing the objective: 9:    $L_{S}=E\left[\log P\left(S=\mathrm{real}|X_{\mathrm{real}}\right)\right]+E\left[\log\left(1-P\left(S=\mathrm{synthetic}|X_{\mathrm{synthetic}}\right)\right)\right]$10:    $L_{C}=E\left[\log P\left(C=c|X_{\mathrm{real}}\right)\right]+E\left[\log P\left(C=c|X_{\mathrm{synthetic}}\right)\right]$11:    $L_{D}=L_{S}+L_{C}$12:  // **Step 2: train generator**13:  Noise *z* and corresponding labels $y_{\mathrm{synthetic}}$14:  Generate synthetic data samples $\tilde{x}=G\left(z,y_{\mathrm{synthetic}}\right)$15:  Update *G* by minimizing the objective:16:    $L_{S}^{\prime }=E\left[\log\left(1-P\left(S=\mathrm{synthetic}|X_{\mathrm{synthetic}}\right)\right)\right]$17:    $L_{C}^{\prime }=E\left[\log P\left(C=c|X_{\mathrm{synthetic}}\right)\right]$18:    $L_{G}=L_{C}^{\prime }-L_{S}^{\prime }$19:**end for**20:Return the trained generator *G*

Based on the proposed method, the performance of a classification model was evaluated on an IoMT dataset by considering three different sets of features: biometric features, network flow metrics, and a combined set of both biometric and network flow features. The primary goal was to determine how each feature set influenced the model’s performance, measured through accuracy, precision, recall, f1-score and confusion matrix, with 10-fold cross-validation employed to mitigate overfitting. First, we analyzed the performance using biometric features, which include physiological signals and other personal biological data (shown in Table 1). The model demonstrated a high accuracy of 96.64%, indicating that it correctly classified the majority of instances. The precision was even higher, at 96.80%, showing that when the model predicted a positive outcome, it was correct 96.80% of the time. The recall was 96.61%, suggesting that the model was effective at identifying true positive cases. The F1-score, which balances precision and recall, was 96.61%, reflecting the model’s overall robustness with biometric data. Similarly, we also evaluated the model using network flow feature sets, which pertain to data derived from network traffic. The model’s performance with these features was slightly lower but still high, with an accuracy of 95.54%. The precision was 95.68%, indicating that the model maintained a good balance between identifying positive cases and minimizing false positives. The recall was 95.50%, showing the model’s effectiveness in detecting true positives. The F1-score was 95.50%, demonstrating a good balance between precision and recall for network flow metrics. The overall evaluations are presented in Table 2.

**Figure 4:**
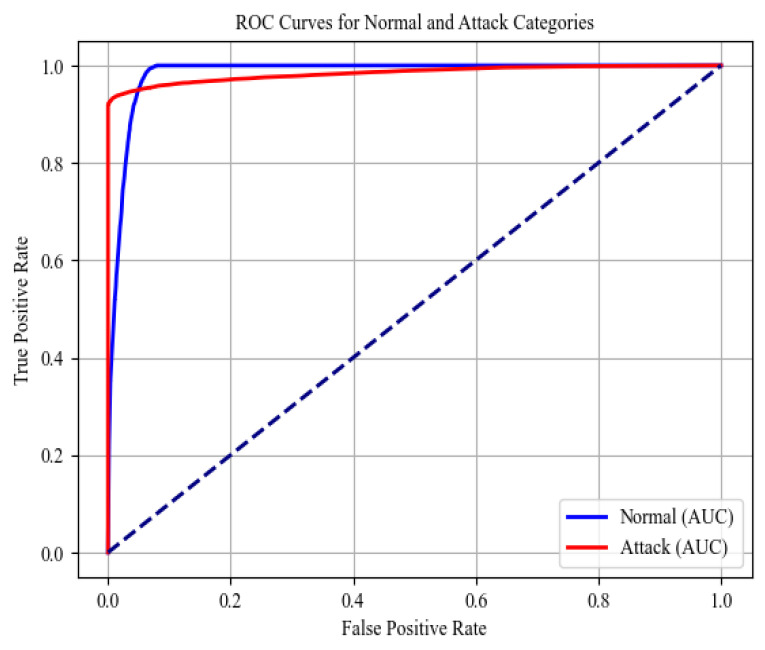
ROC curve of ‘Attack’ and ‘Normal’ category of WUSTL-EHMS-2020 dataset).

Finally, the model’s performance was assessed using a combined set of biometric and network flow feature sets. The combined feature set yielded an accuracy of 96.61%, closely matching the performance seen with biometric features alone. The precision was 96.76%, and the recall was 96.57%, indicating that the inclusion of network flow metrics did not significantly detract from the model’s ability to correctly identify positive cases and minimize false positives. The F1-score was 96.57%, further confirming the model’s balanced performance with the combined feature set. The experimental results showed that the classification model performs exceptionally well across all feature sets. The use of biometric features (eight in total) alone provided the highest individual performance; however, the combined feature set also delivered slightly robust results. The network flow metrics, while slightly less effective, still maintained strong performance metrics. These findings suggest that using biometric data, either alone or in combination with network flow metrics, can significantly enhance the attack detection and reliability of classification models in the context of IoMT datasets. Furthermore, the class label performances (‘Normal’ and ‘Attack’) were examined, presented in the form of confusion matrices for all three scenarios (biometric, network flow, and combined feature sets) in Table 3, Table 4 and Table 5. The receiver operating characteristic (ROC) curve of the combined features is presented in Figure 4. This graphical representation is commonly used to evaluate the performance of a classifier by plotting the trade-off between the true positive rate (sensitivity) and the false positive rate. The ROC curve for the classifier shows a strong discriminative ability, effectively distinguishing between Normal and Attack samples.

## 6. Comparison

**Figure 5:**
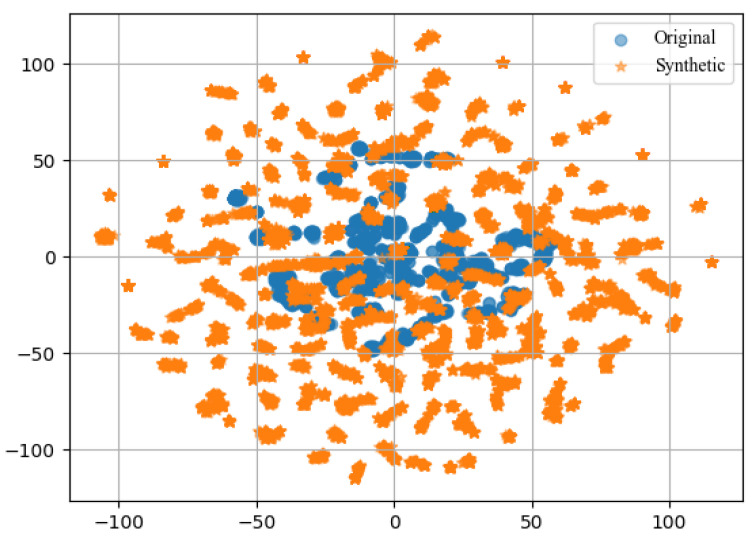
t-SNE visualization of the original attack samples (depicted in light blue) and synthetic samples (depicted in orange) of the EHMS dataset (attack samples).

The EHMS dataset is binary and highly imbalanced, with 14,272 ‘Normal’ samples and only 2046 ‘Attack’ samples, making machine learning models prone to bias toward the majority class [43]. To mitigate this, SMOTE [37] was applied, generating 12,227 synthetic attack samples and thus balancing the dataset with equal numbers of ‘Normal’ and ‘Attack’ samples. However, a key concern is whether these additional synthetic samples accurately represent true attack behavior. To address this, t-distributed stochastic neighbor embedding (t-SNE) was employed to visualize the similarity between the original and synthetic attack samples. The t-SNE plot revealed a high degree of similarity between the real and synthetic attack samples, suggesting that the generated samples closely resemble authentic attack patterns. t-SNE is a dimensionality reduction technique that projects high-dimensional data into a lower-dimensional space, preserving pairwise similarities to provide a qualitative understanding of the dataset’s structure [36]. The t-SNE visualization results are shown in Figure 5.

**Figure 6:**
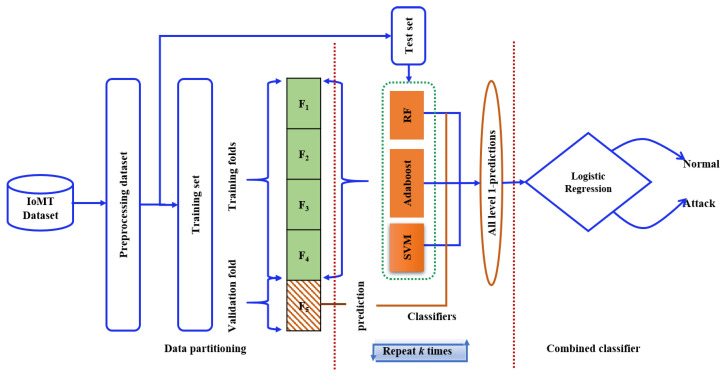
Stack ensemble structure: support vector machine, adaboost, and random forest are base classifiers, and logistic regression is a meta-classifier.

To ensure a fair comparison with the baseline method [1], similar experimental conditions were maintained. Three separate cases were evaluated—biometric features, network flow features, and a combined set of both—with performance measured accordingly. A stack ensemble classifier was implemented, consisting of SVM, Adaboost, and RF as base classifiers, with logistic regression serving as the meta-classifier, as illustrated in Figure 6. This classifier operates in two stages: in the first stage, multiple base classifiers function in parallel, and their predictions are used as input features for the second stage, where logistic regression is applied as the meta-classifier [44,45].

**Figure 7:**
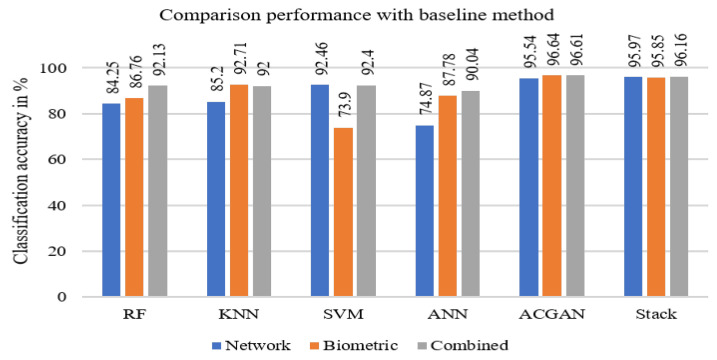
Comparison of the classification accuracies of network flow, biometric, and combined features using different classifiers.

Another goal of this article is to present a comparative analysis of the overall performance of synthetic samples created using SMOTE and ACGAN for an imbalanced IoMT dataset. We also explored other performance metrics, such as precision, recall, F1-score, and confusion matrices, for a more comprehensive understanding of the proposed method. The classification accuracy was consistently higher across all feature sets compared to the original (baseline) method. The baseline method implemented several machine learning algorithms, including random forest (RF), support vector machine (SVM), artificial neural network (ANN), and k-nearest neighbor (KNN), to measure performance. Among these classifiers, RF demonstrated the highest discriminability in the combined feature set, with a classification accuracy of 92.13%. The KNN classifier performed best for biometric features, with an accuracy of 92.71%, while the SVM classifier was most effective for network features, achieving an accuracy of 92.46%. Detailed results are presented in Figure 7. Our results consistently outperformed the baseline across all conditions—biometric, network flow, and the combined feature sets—with accuracies of 96.67%, 95.59%, and 96.61%, respectively. The confusion matrix of stack ensemble of both feature sets is presented in Table 6. The classification accuracies for both classes consistently exceed 95%, as determined through 10-fold cross-validation.

## 7. Conclusions

Security poses a significant challenge for IoT-based HMSs, particularly within the IoMT. Safeguarding patient information, treatment histories, appointments, medication details, and healthcare workers’ data is crucial to ensuring the integrity and confidentiality of healthcare systems. Another critical challenge is finding public datasets of sufficient size for evaluation (training the machine learning model). The EHMS dataset, used for this research, contains 16,318 samples with approximately 87.4% classified as normal and the remaining as attack samples. This imbalance could lead to the machine learning model being biased towards the majority class. To address this issue, we generated synthetic samples that closely resemble the Attack category of the EHMS dataset using an ACGAN. The dataset’s performance was evaluated using two distinct feature sets—network flow and biometric—across three configurations: biometric features, network flow metrics, and a combination of both. Standard performance metrics, such as accuracy, precision, recall, and F1 score, were employed. The results showed that a small set of biometric features (eight in total) had sufficient discriminative ability, achieving a classification accuracy comparable to that of the network flow metrics and their combination. Our results consistently exhibited remarkable consistency and outperformed existing methodologies across all three evaluation scenarios. This experiment demonstrated that biometric features play a critical role in attack detection in IoMT systems.

Furthermore, a stack ensemble classifier was implemented, and SMOTE was applied to generate synthetic samples in the minority class (Attack category). The performance was highly competitive because the stack ensemble integrated different classifiers, which were capable of capturing various attack patterns and improving detection rates. A comparative analysis with the baseline research method used for the EHMS dataset demonstrated the superior performance of the ACGAN approach. High accuracy was achieved for network flow features, biometric features, and their combination, reaffirming the critical importance of effective attack detection within IoMT systems.

The immediate objective was to identify additional IoMT-related datasets that include a wider variety of attack patterns for validation with the proposed model. The EHMS dataset consists of 16,318 samples and is highly imbalanced, with attack samples limited to only two types—spoofing (1124 samples) and data alteration (622 samples)—out of a total of 2046 attack samples. This limited pattern set could restrict the broader attack detection capability of the proposed method. Extending the analysis with datasets containing diverse attack patterns and different Generative Adversarial Networks (GANs) will provide a more comprehensive assessment of the effectiveness of GANs in securing IoMT-related data. Such an exploration would make a significant contribution to improving the attack detection abilities of IoMT, including addressing the challenges posed by attacks.

## Figures and Tables

**Table 1:** EHMS dataset has a set of features divided into biometric and flow matrices: the table shows the feature name, their description, and their types.

Feature	Description	Types
ST	ST segment is the flat section of the ECG	Biometric
Resp_Rate	Respiration Rate	
Heart_rate	Heart Rate	
DIA	Diastolic Blood Pressure	
SYS	Systolic Blood Pressure	
Pulse_Rate	Pulse Rate	
SpO2	Peripheral Oxygen Saturation	
Temp	Temperature	
SrcBytes	Source Bytes	Network flow metric
DstBytes	Destination Bytes	
SrcLoad	Source Load	
DstLoad	Destination Load	
SrcGap	Source Missing Bytes	
DstGap	Destination Missing Bytes	
SIntPkt	Source Inter Packet	
DIntPkt	Destination Inter Packet	
SIntPktAct	Source Active Inter Packet	
DIntPktAct	Destination Active Inter Packet	
SrcJitter	Source Jitter	
DstJitter	Destination Jitter	
sMaxPktSz	Source Maximum Transmitted Packet Size	
dMaxPktSz	Destination Maximum Transmitted Packet Size	
sMinPktSz	Source Minimum Transmitted Packet Size	
dMinPktSz	Destination Minimum Transmitted Packet Size	
Dur	Duration	
Trans	Aggregated Packets Counts	
TotPkts	Total Packets Count	
TotBytes	Total Packets Bytes	
Loss	Retransmitted or Dropped Packets	
pLoss	Percentage of Retransmitted or Dropped Packet	
pSrcLoss	Percentage of Source Retransmitted or Dropped Packet	
pDstLoss	Percentage of Destination Retransmitted or Dropped Packet	
Rate	Number of Packets Per Second	
Load	Load	

**Table 2:** Performance comparison of biometric, network flow, and combined feature sets, measured in terms of accuracy, precision, recall, and F1-score (in %), is presented in Table 2.

Feature Group	Accuracy	Precision	Recall	F1-Score
Biometric	96.64	96.80	96.61	96.61
Network flow metric	95.54	95.68	95.50	95.50
Combined (biometric+network) feature sets	96.61	96.76	96.57	96.57

**Table 3:** Confusion matrix of the biometric feature set presented for each class sample’s distribution percentage.

		Predicted	
		Normal	Attack
Actual	Normal	0.998	0.002
	Attack	0.066	0.934

**Table 4:** Confusion matrix of the network matrix feature set presented for each class sample’s distribution percentage.

		Predicted	
		Normal	Attack
Actual	Normal	0.986	0.014
	Attack	0.076	0.924

**Table 5:** Confusion matrix of the network and biometric feature sets presented for each class sample’s distribution percentage.

		Predicted	
		Normal	Attack
Actual	Normal	0.997	0.003
	Attack	0.067	0.933

**Table 6:** Confusion matrix of the network and biometric feature sets presented for each class sample’s distribution using stack ensemble.

		Predicted	
		Normal	Attack
Actual	Normal	0.97	0.03
	Attack	0.05	0.95

## Data Availability

Data are contained within the article.

## Abbreviations

The following abbreviations are used in this manuscript:

HMS	Healthcare Monitoring System
EHMS	Enhanced Healthcare Monitoring System
GAN	Generative Adversarial Network
IoT	Internet of Things
IoMT	Internet of Medical Things
ACGAN	Auxiliary Classifier Generative Adversarial Network
SMOTE	Synthetic Minority Oversampling Technique
t-SNE	t-distributed Stochastic Neighbor Embedding
EHRs	Electronic Health Records
SpO2	Blood Oxygen Saturation
ECG	Electrocardiogram
WUSTL	Washington University in St. Louis
MAC	Medium Access Control
TP	True Positive
TN	True Negative
FN	False Negative
FP	False Positive
D	Discriminator
G	Generator
SVM	Support Vector Machine
ANN	Artificial Neural Network
KNN	k-Nearest Neighbor
RF	Random Forest
IT	Information Technology
ML	Machine Learning
DL	Deep Learning
AI	Artificial Intelligence
IDS	Intrusion Detection System

## References

[1] Hady A., Ghubaish A., Salman T., Unal D., Jain R. (2020). Intrusion Detection System for Healthcare Systems Using Medical and Network Data: A Comparison Study. IEEE Access.

[2] Jin Y. (2019). Low-Cost and Active Control of Radiation of Wearable Medical Health Device for Wireless Body Area Network. J. Med. Syst..

[3] Li R.T., Kling S.R., Salata M.J., Cupp S.A., Sheehan J., Voos J.E. (2016). Wearable Performance Devices in Sports Medicine. Sports Health.

[4] Bansal A., Kumar S., Bajpai A., Tiwari V.N., Nayak M., Venkatesan S., Narayanan R. (2015). Remote health monitoring system for detecting cardiac disorders. IET Syst. Biol..

[5] Uslu B.Ç., Okay E., Dursun E. (2020). Analysis of factors affecting IoT-based smart hospital design. J. Cloud Comput..

[6] Nguyen T.A., Min D., Choi E., Tran T.D. (2019). Reliability and Availability Evaluation for Cloud Data Center Networks Using Hierarchical Models. IEEE Access.

[7] Bhuiyan M.N., Billah M.M., Bhuiyan F., Bhuiyan M.A.R., Hasan N., Rahman M.M., Miah M.S., Alibakhshikenari M., Arpanaei F., Falcone F. (2022). Design and Implementation of a Feasible Model for the IoT Based Ubiquitous Healthcare Monitoring System for Rural and Urban Areas. IEEE Access.

[8] Zeshan F., Ahmad A., Babar M.I., Hamid M., Hajjej F., Ashraf M. (2023). An IoT-Enabled Ontology-Based Intelligent Healthcare Framework for Remote Patient Monitoring. IEEE Access.

[9] Bhatia H., Panda S.N., Nagpal D. Internet of Things and its Applications in Healthcare-A Survey. Proceedings of the 2020 8th International Conference on Reliability, Infocom Technologies and Optimization (Trends and Future Directions) (ICRITO).

[10] Bathalapalli V.K., Mohanty S.P., Kougianos E., Baniya B.K., Rout B. (2022). PUFchain 2.0: Hardware-assisted robust blockchain for sustainable simultaneous device and data security in smart healthcare. SN Comput. Sci..

[11] Bathalapalli V.K., Mohanty S.P., Kougianos E., Baniya B.K., Rout B. (2022). Pufchain 3.0: Hardware-assisted distributed ledger for
robust authentication in the internet of medical things. Proceedings of the IFIP International Internet of Things Conference.

[12] Verma R.A. (2021). Smart City Healthcare Cyber Physical System: Characteristics, Technologies and Challenges. Wirel. Pers. Commun..

[13] Abdulmalek S., Nasir A., Jabbar W.A., Almuhaya M.A.M., Bairagi A.K., Khan M.A.M., Kee S.H. (2022). IoT-Based Healthcare-Monitoring System towards Improving Quality of Life: A Review. Healthcare.

[14] Philip N.Y., Rodrigues J., Wang H., Fong S.J., Chen J. (2021). Internet of Things for In-Home Health Monitoring Systems: Current Advances, Challenges and Future Directions. IEEE J. Sel. Areas Commun..

[15] Bhardwaj V., Joshi R., Gaur A. (2022). IoT-Based Smart Health Monitoring System for COVID-19. SN Comput. Sci..

[16] Ahmed A., Xi R., Hou M., Shah S.A., Hameed S. (2023). Harnessing Big Data Analytics for Healthcare: A Comprehensive Review of Frameworks, Implications, Applications, and Impacts. IEEE Access.

[17] Tao H., Bhuiyan M.Z.A., Rahman M.A., Wang G., Wang T., Ahmed M.M., Li J. (2019). Economic perspective analysis of protecting big data security and privacy. Future Gener. Comput. Syst..

[18] Wang Z., She Q., Ward T.E. (2021). Generative adversarial networks in computer vision: A survey and taxonomy. ACM Comput. Surv. (CSUR).

[19] Gui J., Sun Z., Wen Y., Tao D., Ye J. (2021). A review on generative adversarial networks: Algorithms, theory, and applications. IEEE Trans. Knowl. Data Eng..

[20] Radford A., Metz L., Chintala S. (2015). Unsupervised representation learning with deep convolutional generative adversarial networks. arXiv.

[21] Odena A., Olah C., Shlens J. Conditional image synthesis with auxiliary classifier gans. Proceedings of the International Conference on Machine Learning.

[22] Peretz D., Arnaert A., Ponzoni N. (2018). Determining the cost of implementing and operating a remote patient monitoring programme for the elderly with chronic conditions: A systematic review of economic evaluations. J. Telemed. Telecare.

[23] Hummel J.P., Leipold R.J., Amorosi S.L., Bao H., Deger K.A., Jones P.W., Kansal A.R., Ott L.S., Stern S., Stein K.M. (2019). Outcomes and costs of remote patient monitoring among patients with implanted cardiac defibrillators: An economic model based on the PREDICT RM database. J. Cardiovasc. Electrophysiol..

[24] Dunbar P., Browne J.P., O’connor L. (2021). Determinants of regulatory compliance in health and social care services: A systematic review protocol. HRB Open Res..

[25] Graafmans W. (2010). EU Actions on Patient Safety and Quality of Healthcare.

[26] Selznick P. (1985). Focusing Organisational Research on Regulation. Regulatory Policy and the Social Sciences.

[27] Bayramzadeh S., Aghaei P. (2021). Technology integration in complex healthcare environments: A systematic literature review. Appl. Ergon..

[28] Alahmar A., Crupi M.E., Benlamri R. (2020). Ontological framework for standardizing and digitizing clinical pathways in healthcare information systems. Comput. Methods Programs Biomed..

[29] Alfian G., Syafrudin M., Ijaz M.F., Syaekhoni M.A., Fitriyani N.L., Rhee J. (2018). A personalized healthcare monitoring system for diabetic patients by utilizing BLE-based sensors and real-time data processing. Sensors.

[30] Taimoor N., Rehman S. (2021). Reliable and resilient AI and IoT-based personalised healthcare services: A survey. IEEE Access.

[31] Schiza E.C., Kyprianou T.C., Petkov N., Schizas C.N. (2018). Proposal for an ehealth based ecosystem serving national healthcare. IEEE J. Biomed. Health Inform..

[32] Lin G., Wen S., Han Q.L., Zhang J., Xiang Y. (2020). Software vulnerability detection using deep neural networks: A survey. Proc. IEEE.

[33] Baniya B., Rush T. (2024). Deep based anomalies detection in emerging healthcare system. Cybersecurity in Emerging Healthcare Systems.

[34] Bhuiyan M.N., Rahman M.M., Billah M.M., Saha D. (2021). Internet of things (IoT): A review of its enabling technologies in healthcare applications, standards protocols, security, and market opportunities. IEEE Internet Things J..

[35] Mazhar T., Talpur D.B., Shloul T.A., Ghadi Y.Y., Haq I., Ullah I., Ouahada K., Hamam H. (2023). Analysis of IoT Security Challenges and Its Solutions Using Artificial Intelligence. Brain Sci..

[36] Joffe E., Bearden C.F., Byrne M.J., Bernstam E.V. (2012). Duplicate patient records–implication for missed laboratory results. Proceedings of the AMIA Annual Symposium.

[37] Zarour M., Alenezi M., Ansari M.T.J., Pandey A.K., Ahmad M., Agrawal A., Kumar R., Khan R.A. (2021). Ensuring data integrity of healthcare information in the era of digital health. Healthc. Technol. Lett..

[38] Ahima (2010). Fundamentals for Building a Master Patient Index/Enterprise Master Patient Index (2010 Update).

[39] Zan S., Agboola S., Moore S.A., Parks K.A., Kvedar J.C., Jethwani K. (2015). Patient engagement with a mobile web-based telemonitoring system for heart failure self-management: A pilot study. JMIR MHealth UHealth.

[40] Baniya B.K., Rush T. Intelligent Anomaly Detection System Based on Ensemble and Deep Learning. Proceedings of the 2024 26th International Conference on Advanced Communications Technology (ICACT).

[41] Baniya B.K. Intrusion Representation and Classification using Learning Algorithm. Proceedings of the 2022 24th International Conference on Advanced Communication Technology (ICACT).

[42] Mirza M., Osindero S. (2014). Conditional generative adversarial nets. arXiv.

[43] Liu S., Wang Y., Zhang J., Chen C., Xiang Y. (2017). Addressing the class imbalance problem in twitter spam detection using ensemble learning. Comput. Secur..

[44] Tang J., Alelyani S., Liu H. (2015). Data Classification: Algorithms and Applications. Data Mining and Knowledge Discovery Series.

[45] Wolpert D.H. (1992). Stacked generalization. Neural Netw..

